# Criminalized Black Women’s Experiences of Intimate Partner Violence in Canada

**DOI:** 10.1177/10778012211035791

**Published:** 2021-09-21

**Authors:** Patrina Duhaney

**Affiliations:** Faculty of Social Work, 2129 University of Calgary, Calgary, Alberta, Canada

**Keywords:** intimate partner violence, domestic violence, Black women, victimization, criminalization, critical race feminism

## Abstract

Canadian research examining the overlap between Black women's victimization and criminalization is sparse. This qualitative study addresses this gap by examining the ways in which criminalized Black women's intersecting identities of race, class, and gender influence how they perceive, experience, and respond to intimate partner violence (IPV). Semi-structured interviews were conducted with 25 Black women who experienced IPV. The findings focus on the women (15) who were also charged with an IPV-related offense. Critical race feminism was employed to analyze their narratives. This research has implications for policy, practice, and future research with Black women who are victimized and criminalized.

## Introduction

Intimate partner violence (IPV) is a complex and persistent public health issue that disproportionately affects women (Burczycka, 2018; Sinha, 2013). In 2018, there were over 99,000 victims (aged 15–89 years) of police-reported IPV incidents in Canada, involving individuals who were legally married, common law spouses, and those in dating relationships (Conroy et al., 2019). Compared to their male counterparts, women (79%) were overrepresented as victims of IPV (Conroy et al., 2019) and were more likely than men to be victims of sexual violence (Cotter & Savage, 2019). The consequences of IPV are more severe for women who experience a range of negative mental and psychological outcomes (i.e., depression, anxiety, fear, low self-esteem) (Ansara & Hindin, 2011; Hutchins & Sinha, 2013), sustain injuries (Sinha, 2013), and are killed more frequently by an intimate partner (Conroy et al., 2019; Sinha, 2013).

Through ongoing advocacy, largely from grassroots organizations, there has been significant strides to fight and eliminate violence against women (VAW). These include increased awareness of IPV and a proliferation of governmental and institutional responses including the development of several government initiatives, establishment of various VAW organizations and supportive services for victims of abuse across Canada (Moreau, 2019). Mandatory charging and prosecution policies were also adopted to provide the police and crown prosecutors with charging and prosecutorial authority, remove the responsibility from victims to lay charges, increase reporting of assaults, and reduce reoffending (Department of Justice, 2017). The implementation of these policies has had significant consequences for women. There has been a substantial increase in the number of female victims charged and criminalized following police intervention, giving rise to considerable debate among scholars about the extent to which women engage in reciprocal violence in their relationships (Hamberger & Larsen, 2015). Feminist researchers argue that women are predominantly victims of IPV (Dobash & Dobash, 2004), whereas some family violence researchers argue that women perpetrate violence at equal rates as men (Fiebert, 2014; Straus, 2010). Despite these ongoing debates, the victimization rate among women has remained relatively high (Burczycka et al., 2018).

There has been extensive scholarship exploring the frequency and extent to which violence occurs in intimate relationships (Black et al., 2011; Burczycka et al., 2018; Sinha, 2013; World Health Organization, 2021), however, much of this research focuses primarily on the experiences of white women and men (Ristock, 2011; Taft et al., 2009). There is growing concern among scholars that racialized women face unique vulnerabilities that increase their risk of IPV (Abraham & Tastsoglou, 2016), yet their experiences continue to be largely homogenized. Specifically, within the Canadian context, all racialized women are classified as visible minorities. Advocates and organizations have emphasized the significance of collecting race-based data (Alliance for Healthier Communities, 2020), however, the Canadian government has yet to collect and publish disaggregated data that shows the prevalence of IPV among Black or other racialized groups. The absence of data renders Black women invisible and makes it difficult to assess their unique experiences of IPV and its consequences in their lives. However, comparisons can be made with Black women in the United States, given that both groups face deeply entrenched systemic racism and various social, economic, and political barriers (Creamer, 2020; Statistics Canada, 2020a, 2020b). These realities are further informed by a growing body of U.S. research that documents Black women's differential experiences of IPV and the ways in which social inequalities have shaped their experiences (Bent-Goodley, 2004, 2011; Gillum, 2019; Nash, 2005; Potter, 2008; Richie, 2012; West, 2007).

This study aims to address the gap in Canadian IPV research by exploring the complexities and cumulative effects of violence in Black women's lives. It illuminates the connections between their intersecting identities (i.e., gender, race, class) and experiences of victimization and subsequent criminalization.

## Literature Review

### Black Women's Experiences of IPV

Existing U.S. data show that the prevalence rate of IPV among Black women and men is disproportionately high (Black et al., 2011; Truman & Morgan, 2014). Some researchers have argued that socio-economic factors explain differences in IPV rates among racial groups (Rennison & Planty, 2003; West, 2004). Rennison and Planty (2003) found that IPV was more significant along income rather than racial lines for both Black and white victims. Specifically, Black, and white couples with lower annual household incomes experienced IPV at a higher rate than those with higher annual household incomes. These findings are not surprising given that a recent Statistics Canada report showed that Black adults are less likely to be employed than the rest of the population (Statistics Canada, 2020a). Other structural factors (e.g., educational inequality, institutional racism) (Gillum, 2019; Hampton et al., 2003) have been shown to increase Black women's risk of IPV (Carney & Buttell, 2006; Carbone-Lopez & Kruttschnitt, 2010). In a recent Ipsos poll that surveyed 1,000 Canadians, 54% of respondents believed that racism is deeply ingrained in the Canadian economy, government, and educational system (Bensadoun, 2021). These findings suggest systemic and institutional racism create unique challenges for Black women which affect various aspects of their lives. Likewise, situational contexts (e.g., substance use) impact the risk and perpetration of violence. In their study that examined the association between IPV and different types of substances, Nowotny and Graves (2013) found that when one or both partners used substances there was an increase in the likelihood and severity of various forms of IPV perpetration and victimization. They found that Black women may use substances as a coping mechanism for different stressors in their lives.

Black women who experience IPV must also confront societal, cultural, and familial cues that silence them from speaking up about their experiences of violence (Potter, 2008; Stockman et al., 2014). Consequently, to protect themselves and their partners from further discrimination (Nash, 2005), they may be ambivalent about seeking support from social service providers (Ullman & Lorenz, 2020) and the criminal justice system (Goodmark, 2008). According to Bent-Goodley (2001), “this reluctance is often a reflection of feeling left out of the formal system and a learned behaviour of self-survival” (p. 322). Black women's experiences of IPV are further compounded by stereotypical images and caricatures typifying them as strong, angry, aggressive, and violent. These images conflict “with the notion of the passive, helpless battered woman” (Harrison & Esqueda, 1999, p. 133) that is often used to characterize white women. Stereotypical images that construct Black women as aggressive or violent are problematic as they may also prevent them from being considered credible victims in IPV incidents (Brown, 2012). Consequently, these pervasive stereotypical images reinforce the belief that Black women are capable of overcoming challenges on their own.

Black women's experiences of IPV are also confounded by sexism, racism (Richie, 2012; Taylor, 2005), and immigration status (Lacey et al., 2021), which can impede their decision to terminate an abusive relationship. Moreover, the lack of resources available to them limits their options for escaping violence (Gillum, 2008). Still, some women rely on internal supports such as family and friends (Few, 2005; Gillum, 2008; Taylor, 2008) and utilize faith-based or religious facilities as a coping mechanism (Bent-Goodley & Fowler, 2006; Potter, 2007a). Nonetheless, informal supports may be insufficient on their own to counter the effects of male partner violence and various forms of oppression. Black women who believe that they have limited options when terminating an abusive relationship may fight back to resist violence and protect themselves (Richie, 2012). As West (2007) notes, Black women's use of force occurs within the context of their own victimization. However, the overlap between their victimization and use of force in their intimate relationships is often blurred by economic and structural marginality and oppression on multiple levels. Richie (1996) describes the socially constructed processes (i.e., persistent poverty, violence, and an overly punitive criminal justice system) that lure and entrap Black women as gender entrapment. Moreover, these women are often “penalized for behaviors they engage in even when the behaviors are logical extensions of their racialized gender identities, their culturally expected gender roles, and the violence in their intimate relationships” (Ritchie, 1996, p. 5). Taken together, it is necessary to discuss how cumulative and multiple traumatic experiences of IPV entrap criminalized Black women and deprive them of meaningful options to escape male partner violence.

### Theoretical Framework

Critical race feminism (CRF) is the theoretical approach that informs this study and helps to conceptualize and deepen understanding about the relationship between Black women's victimization and criminalization. CRF is relevant to my analysis because it acknowledges that Black women's experiences have merit (Wing, 2003) and forefronts issues of race, racism, power, and various other forms of oppression (Solorzano & Yosso, 2002). It also illuminates the multiplicity and intersectionality of Black women's identities (Crenshaw, 1991).

Consistent with the primary tenets of critical race theory, CRF proponents recognize that racism is an everyday occurrence deeply ingrained in society (Razack et al., 2010). Because it is perceived as ordinary and natural, laws or rules that encourage treating everyone equally obscure racism (Matsuda, 1989) and do very little to address covert racism or the everyday alienation, despair, and invisibility that racialized people encounter (Brown, 2007). CRF scholars draw on an intersectional lens to analyze the ways in which Black women's experiences of IPV are compounded by multiple and overlapping forms of oppression, such as race, class, and gender, histories of exclusion and disadvantage, poverty, unemployment, and immigration status (Crenshaw, 1991; West, 2005). Critical race feminists argue that the law is not neutral and that the social disadvantages experienced in society often contribute to racial disparities in the criminal justice system. According to Chan and Chunn (2014), “systemic and institutional discrimination is a key factor in the differential treatment of racialized people” (p. xiv). Furthermore, “racial prejudice has contributed to the dismissal and/or minimization of criminal victimization involving racialized people … and has perpetuated the view that certain racialized groups in Canada are more prone to crime” (Chan & Chunn, 2014, p. xiv). Given Black women's history of social disadvantage and oppression, it is necessary to advance theorizing that unequivocally positions Black women's experiences of IPV at the forefront of the discussion while highlighting how these experiences are complicated by intersecting and overlapping forms of oppression. Finally, CRF is a viable framework because it allows me to present women's counter-narratives that help disrupt myths and negative constructs that misconstrue their realities.

I use the term criminalized to refer to Black women who have been arrested to “signal processes and practices rather than a reified identity … and to challenge discourses that construct criminalized women as ‘other’ and separate them from those who research and write about their experiences” (Pollack, 2007, p. 172). Using the term criminalized to characterize Black women's use of force allows me to “place women's violence within the social context in which it occurs … it does not excuse the behaviour, but it does assist in making it more understandable, especially in terms of the choices available to these women” (Comack & Brickey, 2007, p. 27). Black women who have had a long history of victimization and defend themselves against an abusive partner are criminalized for their actions, which leads to harsh and punitive treatment in the criminal justice system. This article addresses two research questions: (a) What are Black women's experiences of IPV? and (b) How do Black women's intersecting identities of race, class, and gender influence how they perceive, experience, and respond to IPV?

## Method

Participants were recruited between June 2018 and January 2019, following ethics approval from the Research Ethics Board at Wilfrid Laurier University. Two techniques were used to recruit participants, purposive sampling and snowball sampling. Purposive sampling involves selecting participants based on certain characteristics and who can provide great insights about a phenomenon or area of inquiry (Creswell & Creswell, 2018). Recruitment flyers were posted at agencies and organizations across the Greater Toronto Area (GTA) and surrounding regions providing services to women who are victims of IPV or who were charged with an offense. Flyers were posted in grocery stores, restaurants, churches, health, and beauty supply stores. Information about the study was also shared on community boards, libraries, listservs, professional websites, and social media (Twitter, Facebook, Eventbrite). Through snowball sampling, research participants were asked to nominate someone else whom they believe would be interested in participating in the study. Professional contacts were also solicited for assistance in reaching potential participants.

The criteria for inclusion in the study included the following: (a) age 18 years or older; (b) experience of IPV; (c) charged or arrested by the police; and (d) involved in a heterosexual relationship at the time of arrest (it was necessary to recruit these women to better understand the dynamics that unfold in heterosexual relationships). Informed written consent was obtained from each participant prior to their interview. Participants completed a demographic questionnaire that asked questions about their race, ethnicity, age, employment status, income, level of formal education, and religious affiliation. These questions were meant to inform understanding of how intersecting aspects of Black women's identities influence their experience with IPV. Semi-structured interviews were conducted with participants in a private room at a nearby public library or in their homes and lasted for approximately two hours. Each participant was assigned a pseudonym to protect their identity. Participants were asked to discuss the following questions: (a) Have you experienced abuse in a previous or current relationship? (b) Could you please tell me more about this experience? How often did the abuse occur and how long did it last? (c) How has race affected your response to abuse in your intimate relationship? (d) How has your gender or class influenced your responses to abuse in your relationship? (e) In what ways have external factors such as pressures at work, loss of employment, and so on influenced your experience of abuse?

Participants received an honorarium of $35 as a small token for their involvement in the study. Interviews were audio-recorded, then transcribed shortly after meeting with participants. Coding and analysis of the data involved three stages. In the first stage, transcribed interviews were read to uncover preliminary themes. In the second stage, interviews were uploaded to NVivo 12 for MAC, a computer software program that supports qualitative and mixed methods (QSR International, n.d.), to further explore themes in greater depth. In the third stage, recurring themes were conceptualized using CRF. These themes assisted in describing the relevance of race, racism, and power in Black women's lives, as well as the overlap between their victimization, criminalization, and how various aspects of women's identities and experiences are influenced by oppression and social marginalization.

## Findings

### Sample Characteristics

The full sample in this study was composed of 25 Black women who were victims of IPV in a heterosexual common law or marital relationship. Fifteen of the 25 women reported that they were also charged with an IPV-related offense following police contact; these women are the focus of the article. Four of the women were in a relationship with a white man, one woman was in a relationship with a man of mixed race, and 10 women were in a relationship with a Black man. Women ranged in age from 29 to 57 years old with an average age of 39 years and lived in the GTA and surrounding areas. Five of the women were born in Canada, eight in the Caribbean, one in South America, and one in Africa. Seven of the women had completed high school, seven had a college diploma, and one had a university degree. The women's annual household income ranged from less than $24,999 to $100, 000 or more; the majority (11) of the women had incomes of less than $35,000 a year.

Following police intervention, 13 of the 15 women were charged as the primary aggressor (their partners were not charged) and two of the women were dually charged alongside their partners. Three women disclosed that they had previous contact with the police in which the women were the accused, but only one of these women indicated that her previous police encounter was related to a domestic incident. Charges against the women included uttering threats, assault, and assault with a weapon. All the women were charged with at least one count of assault.

The next section highlights five main themes that emerged from the women's interviews: (1) violence in Black women's lives; (2) influence of substances; (3) economic constraints as an aggravating factor; (4) stereotypical images of Black women; (5) fighting back as a form of resistance.

### Violence in Black Women's Lives

During the interviews, the women disclosed the many forms of male partner violence they had experienced, ranging from verbal, emotional (e.g., humiliation, degradation), psychological, physical (e.g., kicking, choking, slapping, punching, and biting), sexual, and financial abuse. One of the women in the study, Desiree, disclosed that she experienced emotional and psychological abuse daily, which she began to internalize. She shared that,

At first it was verbal abuse, bitch, slut, whore, every day. And then emotionally. I started accepting. … He was always putting me down, the way I walk, the way I talk, the way I dress.

Similarly, Kiara described being in an abusive marriage where she was denigrated. She stated,

In my marriage I felt like I was nothing … and I was depressed, I didn't talk about it. It didn't help me because my husband made me feel like I’m not worth anything. He made me feel like he was enough, my life stopped there.

Jocelyn also encountered emotional and psychological abuse in her marriage. She said her husband berated her to the extent that she considered harming herself:

He can insult you in a way you feel like killing yourself, I swear. I don't know how to say the words he says … this makes me so angry. I raise my voice, and from there he gets angry. He would use words like I’m "a nobody," I’m "an idiot," I’m "useless, slutty cunt.”

Emotional abuse was one of the many forms of violence that characterized Black women's lives. The manifestation of this violence in their lives contributed to feelings of insecurity, inadequacy, disempowerment, and entrapment. Women's narratives underscored the devastating short- and long-term effects of psychological abuse. These incidents of violence from their partners worsened over time and increased their risk for more severe forms of violence.

Except for one woman, all study participants shared that they had been physically assaulted by their husbands or common-law partners. Many women were injured during these assaults. Pamela was married at a young age and had four children with her abusive husband. The violence was so severe that she was forced to leave her children behind in the Caribbean to escape. She conveyed:

I got beat up a lot with my first husband. He didn't know what he wanted and at 16 years old, I didn't even know what I wanted. He used to beat me up a lot and I pray to God, and I tell him I want to come abroad, and I did, so I can leave him behind. I leave my kids there. That's when I realized that I did something terrible, leaving my kids behind.

Pamela's story captured the severity of the violence to which some of the women were subjected. With little support from her family, she felt she had no choice but to flee to safeguard against further violence.

A few of the women disclosed that they had been sexually abused in their relationships. Women shared that they were coerced into having sex with their partners; some believed the sexual violence perpetrated by their partners were attempts to gain dominance and control over them. Dixie reflected on being coerced into having sex with her intimate partner while they were going through a divorce. She said she made it clear that she had no interest in him, but he ignored her wishes and raped her. She asserted,

I was trying to get out of it. And the feeling of being raped by your husband is like the worst thing in the world. I always felt like he did that on purpose as a control thing. That was his abuse.

Most women who experienced sexual violence, in particular marital rape, did not report these incidents to formal supports or the police for a variety of reasons, including fear they would not be believed. Shortly after moving in with an older man, Gabby said she developed a friendship with him which later evolved into an intimate relationship. She expressed having financial difficulties at the time and was forced to engage in a sexual relationship with him to receive financial support. Gabby did not report her experience to the police because she believed the age difference between her and her partner would reflect negatively on her. Gabby stated,

Not that I wanted to have a sexual relationship with him but, you know, he was the one in charge of everything. It was his house. Everything was under his name. Yeah, he can't give me money for free. … He's an older guy, I’m 30 plus and um, they might think I’m up to no good, you know.

### Influence of Substances

For some women, the presence of drugs and alcohol increased their risk of severe forms of violence. Injuries sustained ranged from minor (e.g., scratches, bruises) to severe (e.g., broken bones, head injuries, internal injuries). Evelyn chronicled an incident with her partner in which they were both under the influence of drugs and alcohol. She explained that she was thrown from the hood of his car:

He got physically aggressive with me and started throwing alcohol bottles at me. He almost split my face open and then we tussled on the ground. I went downstairs and I was going to take my shit and leave. Then he came downstairs, he turned the car on. I’m on the hood of the car. He swung so fast I almost broke my arm. Then he drove off with my wallet and everything in the car and I’m like, “Oh my God, what do I do now?” I’m like, “I have no choice!”

Crystal described the injuries that were inflicted by her partner:

I’m always getting hurt. As I said, he will knock me in the shoulders, the head, the side. I’ve had pictures with my side bruised up all the time. … I guess I just got used to it. … The worst was the side because the tummy and the side always hurt.

The frequency and severity of violence in Black women's lives coupled with the influence of substances have deleterious consequences for Black women. Yet, due to various aggravating factors, many women were reluctant to seek support.

### Economic Constraints as an Aggravating Factor

Economic constraints further amplified the IPV perpetrated against Black women and increased their vulnerability to subsequent criminalization. In a few instances, women shared that they were the primary breadwinners because their partners were either unemployed or did not receive enough income to adequately provide for their families. Dixie worked so that she could look after the family. However, conflicts arose in the relationship when her partner refused to help with responsibilities at home. As she stated,

There was a time when he wasn't working, but he insisted that the baby should still go to daycare, which was stupid because it was costing more money than we could afford. But he didn't want to look after her. … I had to get up, get the baby ready, take her to daycare at 6 o’clock in the morning, or 7 o’clock. … And then the next one came … and he was getting progressively more abusive, hitting and stuff like that, and extremely antisocial.

Based on Dixie's statement, it is possible that her work outside of the home disrupted the traditional gendered division of labor where the husband was primarily responsible for the family's economic stability; this may have contributed to her husband's retaliatory actions. Jocelyn also expressed that she and her husband argued frequently over money. At the time they were saving for a house, but he was not forthcoming with his earnings. Jocelyn shared:

We had a disagreement about money … and that was what aggravated the quarrel. We were saving money for the house … and I was like, “Okay, let me see what you’re splitting so I know I’m getting my fair share.” And he didn't used to show me, and that's what escalated the whole thing. He didn't want to. … He actually slapped me across the face.

Janice also experienced economic marginalization; however, it was further compounded by her immigration status and precarious employment. She met her partner while he was visiting the Caribbean. After a few years of dating, they got married and she immigrated to Canada. However, her husband started displaying erratic behavior shortly after her arrival. The little money he received from his pension was spent mostly on alcohol. She said he only gave her $100 for the entire month to buy groceries. She expressed:

I said, you know, “The time you would take money to buy all this booze, you can buy food for us to eat.” Like, it was really a struggle. So, I started braiding hair at home and then the money that I would get … I would buy food and then he would demand that money from me. Then I started standing up. I said, “No.”

### Stereotypical Images of Black Women

Black women's experiences of IPV are further complicated by pejorative stereotypes that depict them as angry, loud, aggressive, and violent (Collins, 2000). Likewise, another problematic characterization is that of the strong Black woman, which has both positive and negative attributes as it depicts Black women as both assertive and self-sacrificing (Etowa et al., 2017; Watson & Hunter, 2016; West, 2012). These stereotypes have significant consequences for Black women. A few of the women in relationships with white men shared that they did not believe they would be viewed favorably if they reported the abuse. For these women, Black women may not be perceived as credible victims. The women's narratives reflect some of the stereotypes that framed their experiences.

During our interview, Gabby shared that her neighbors overheard frequent arguments between her and her partner. From her encounters with some of her neighbors, she suspected that they perceived Black people, particularly Jamaicans, negatively. Her remark captures one of the most persistent stereotypes that Black women encounter daily, one that constructs them as loud. As she stated,

“Well, nothing is wrong with being Black, but you know they just stigmatize against Black … ‘She's from Jamaica and she is very loud’ and stuff like that.” It is possible that Gabby's neighbors normalized the frequent arguments between her and her partner as something that is common among Black couples.

In the following statement, Natalie articulates the negative constructs she believes characterize the relationship between Black women and Black men.

They think that Black guys, all they do is get girls pregnant, they don't take care of their children. They beat up their women because Black women are very stubborn, and they don't listen. Um, let's see what else, that Black girls are sluts, you know, it's their fault. They’re very mouthy, they have no respect and, you know, they don't know how to carry themselves as women. And so therefore a man has the right to, you know, show them their place. That's what I believe.

Natalie's narrative pointed to a widespread assumption about the absenteeism of Black fathers and their disregard for their familial responsibilities. The other assumption relates to the “angry Black woman” construct. Those who accept these negative racialized and gendered stereotypical assumptions, as Natalie's story demonstrates, are more likely to perceive that Black women are responsible for their victimization and believe that violence against them is justified.

Another stereotype that is often used to characterize Black women is that of the “strong Black woman,” which was endorsed by more than half of the women. The strong Black woman construct is both a stereotype and a reality in Black women's lives (Potter, 2008). According to Ruth,

“Black women tend to portray that they’re big and strong, that nobody can touch them. And some of them really come across really angry.” For Serena, being strong is a necessary survival mechanism that she was taught at a young age.

Well, I mean just deal with it. You don't sit there and cry in your Cheerios, get over it. You know what I mean? You don't get depressed. You don't sit there. You just deal every day. It's just how culturally you’re taught, be strong, don't be a punk.

Some women believed they had no option than to be strong because they did not have the same protection as white women. As Janice shared,

I just feel like we have no rights. I don't feel like we have rights. … It is racism. … I struggle with that even at work. It's everywhere.

Women who endorsed the strong Black woman stereotype were more likely to remain silent about the abuse they experienced. As Crystal stated:

I was ashamed when a lot of things started happening. So, for me, keeping it private was my best solution because if nobody knew about it and I try to fix it myself, thinking that I was doing a good thing, that was my comfort. But the pain became real, you know what I mean? It's different when we’re trying to do things ourselves but when the pain became real for me, being private wasn't enough for me anymore.

Women may be reluctant to share future incidents of abuse with others if their experiences are minimized or considered inconsequential. For example, some women shared their experiences with others, but they were encouraged to remain silent. According to Jocelyn, both her mother and her partner's mother were aware of conflicts in her relationship, but they encouraged her to try to cooperate with her partner. She expressed:

I spoke to my mom, and his mom knew he could be like that. And they’ll be like, “When he starts to insult you or call you names, try and keep quiet and don't say anything. Just be mute, and don't let it escalate,” and stuff like that. But they don't hear what he calls me. They don't hear him yell and call me names.

Black women in this study discussed being described as strong willed, outspoken, and assertive. Endorsement of this stereotype also has implications for how women are treated by their partners, family members, the criminal justice system, and society. For example, Black men who hold these stereotypes may rationalize negative treatment of Black women. Stereotypical images of Black women also constrained many women in this study to remain silent about their abuse and contributed further to their isolation.

### Fighting Back as a Form of Resistance

Black women engaged in various forms of resistance against their abusive partners. However, the cumulative effects of violence along with structural and community contexts motivated some women to physically fight back against their partners to protect themselves and resist subsequent violence.

A few women shared that they had witnessed their mothers being abused and did not want to have a similar experience. As Candice acknowledged,

I was watching my mom being abused and I wasn't going to be abused. I don't even understand how that happens, but I think it has something to do with my mom being abused.

Like other women in the study who were abused by their partners, Crystal attempted to resist further violence by fighting back.

We got into an altercation. … I don't remember what he used to hit me … as soon as he hit me with it, that was it. … I hit him back because … I’m not going to take any abuse from nobody. So, we rolled … and we rolled, and fight and I fought.

For Jocelyn, fighting back was necessary to stay alive:

I was responding to him hurting me because he was so abusive. He's really big. I don't want to die with him hitting me. And then I tried to fight back. I’m like, “Don't touch me. Don't hit me.” He hit me; I would hit back. Sometimes he laughs, and he's like, “You think you can fight me?” I was like, “Even if I die trying, I will still try.”

Although Gabby's partner was physically stronger than her, she disclosed that when he abused her, she had to fight back:

When my partner started abusing me, I don't just sit there and take abuse because I will curse back and abuse back. … I have to fight back, you know, because I have to stand up for my rights, too. It's impulsive, you’re in the abuse.

The women's narratives provide great insights into the many factors that contribute to their use of defensive tactics against an abusive partner. However, the barriers and consequences that followed had severe and long-lasting negative effects in their lives as some women lost their children, their homes, and their jobs, and had to abide by court directives.

## Discussion

The present study sought to examine criminalized Black women's experiences of IPV in Canada and the factors that influenced their responses to IPV. Consistent with findings from Potter's (2008) study, Black women experienced high levels of physical forms of violence that intensified during their relationship. Like Richie’s (2012) study, results showed that women's partners deployed “a set of psychological, emotional, and/or verbal tactics that result in fear, anxiety, or … confusion” (p. 32). Black women in this study were also subjected to sexual violence in their relationships. Comparable findings were reported by Black et al. (2011), who found that a disproportionate number of Black women experienced sexual violence (i.e., rape) at some point in their lives. Most women did not disclose their experience of sexual violence to formal supports. Black women's discomfort and reluctance to discuss their experiences of sexual violence are “inextricably linked to gender and race. … [Black women are] judged as less truthful and more to blame for their rapes” (Donovan & Williams, 2013, p. 180). Black women's nondisclosure of forced sex by their husbands or common-law partners is further compounded by the perception that their sexual encounters are consensual. Since these encounters have historically been disregarded as rape (Randall & Venkatesh, 2015), there is increased motivation for women to remain silent.

The use of substances increased the likelihood of violence against Black women. Indeed, there is substantial evidence that documents the relationship between substance use (i.e., alcohol, drugs) and violence (Cafferky et al., 2016; Carbone-Lopez & Kruttschnitt, 2010; Carney & Buttell, 2006; Stuart et al., 2008). It was apparent from the women's narratives that the presence of drugs and alcohol increased the frequency and severity of violent victimization in their relationships. Although a couple's use of substances in their relationship may increase their risk of violence (McKinney et al., 2010), Black people may be more susceptible due to their histories of racial oppression and social and economic marginalization. In their study, Stevens-Watkins et al. (2012) found that Black women's lower socio-economic status and experiences of racism increased use of substances for some of the women.

The intersections between gender, race, and class were interwoven with women's economic marginalization and their risk of IPV. Women with greater economic marginalization were more likely to report experiencing IPV. This is not surprising given the precarity of employment for Black people in Canada when compared to their white counterparts (Cranford et al., 2003). Moreover, frequent disagreements over money or the distribution of funds are not uncommon for people who are economically marginalized. Gendered expectations and Black men's desire to provide for their families necessitates economic independence; however, their attempts are often hindered by racism and systemic barriers (i.e., economic disadvantages). According to Hampton et al. (2003), “the situational context in which intimate partner violence occurs among African Americans is, in many ways, a product of the various structural forces (e.g., institutional racism, cycles of chronic underemployment and unemployment, poverty, etc.)” (p. 542). Similarly, Chaney (2009) states, “racism greatly minimizes the likelihood that Black men can educationally and economically be independent and stand on their own” (p. 119). It is possible that these structural barriers impacted Black men's ability to fulfill their responsibilities to adequately provide for the family which worsened the family's economic challenges and contributed to elevated relational conflicts (e.g., anger, frustration). Consequently, these conditions created unpredictable and violent circumstances for Black women.

Another factor that increased Black women's vulnerabilities to violence was pejorative stereotypes. Findings from the current study suggest that stereotypical images that portray Black women as strong, angry, aggressive, or violent complicate their experiences and responses to IPV and may contribute to negative consequences in their intimate relationships. Many of the participants endorsed the strong Black woman construct. For these women, there was an expectation that they should be strong enough to endure the abuse and resist an abusive partner; these beliefs are well supported in the scholarship (Richie, 2012). As Goodmark (2008) states, “victimhood is intimately tied to traditional notions of womanhood, notions that have been largely defined by a white norm … this construction deprives Black women of victim status and its associated protections” (pp. 85, 86). West (2004) also highlighted the historical significance of negative portrayals of Black women and maintained that these portrayals have been used to normalize violence against them. These negative constructs have negative consequences for criminalized Black women who experience IPV. Gillum’s (2002) study alluded to the negative consequences of stereotypes. Based on her findings, 71% of the Black men she interviewed endorsed the matriarch stereotype (strong Black women). Black men who endorsed these stereotypes were more likely to use violence against Black women. She cautions that endorsing stereotypical images of Black women is one of the many factors that negatively affects relationships between Black women and men. Nonetheless, these pejorative stereotypes coupled with systemic anti-Black racism may contribute to beliefs that Black women are not credible victims. Comparable findings were captured in Esqueda and Harrison’s (2005) study, which found that of the 288 white participants (138 women and 150 men) interviewed, most participants believed that Black women were more culpable for IPV than white women. The authors believed that biases based on race and gender influenced participants’ perceptions of Black women.

In response to the harmful and cumulative effects of violence in their lives, some Black women physically fought back against their abusive partners. Fighting back is used broadly to conceptualize Black women's resistance and reflects their “active capacity to oppose, avoid, and push back against the abuse and its negative effects, the abuser and abusive relationships, and the broader social environment that upholds social and cultural norms of violence against women” (Crann & Barata, 2016, p. 860). Although previous research shows gender symmetry in IPV perpetration (Fiebert, 2014; Straus, 2010), women did not believe their use of force was comparable to their partners’ violence in terms of frequency or severity. These results reflect those of other researchers (Moe, 2004; Moss et al., 1997; St. Vil et al., 2017), who also found women fought back and used force to prevent further abuse. In addition, except for a few women, women did not believe they used force to exert power and control over their partners; these findings contradict previous research (e.g., Kernsmith, 2005; O’Leary & Slep, 2006; Ward & Muldoon, 2007). It is important to highlight the ramifications for Black women who use force against their partners. According to Goodmark (2008), “women who fight back undermine societal assumptions about appropriate gender roles and how a battered woman should respond to violence” (p. 94). As indicated previously, of the 15 women who were arrested, 13 were charged solely with perpetrating violence, which reflects the negative outcome of pro-charging laws that criminalize victims of IPV. Nonetheless, these findings also allude to the overlap between women's victimization and criminalization, which are not mutually exclusive categories. By highlighting the complexities of violence in Black women's lives, we gain a better understanding of the various factors and circumstances influencing their use of force and their responses to violence in their relationships.

### Limitations and Strengths

Several limitations were evident in the current study. Gaining access to a large sample of women was difficult due to several factors including fear and distrust. A larger sample would provide additional insights. Another limitation was related to study criteria; due to practical constraints, this study focused solely on women who were English speaking. Language barrier concerns could be addressed by using interpretation services to increase accessibility to women who do not speak English. Due to the nature of the study, it was not possible to determine the extent to which women's immigration status increased their risk of victimization or criminalization. The study was based on self-report data. Given the stigma associated with violence and criminality in Black communities, it is possible that some women may understate the gravity of these issues. There may also be variations in how women interpret interview questions. Despite the limitations noted, this study has several strengths. The paucity of Canadian data has made it difficult to understand the complexities of violence in Black women's lives. This study is one of a few Canadian studies that explore diverse perspectives of an ethno-culturally diverse group of Black women. It examines Black women's experiences of IPV and the overlap between their victimization and criminalization. It also provides further evidence that contextualizes and complicates Black women's use of force in their intimate relationships.

## Conclusions and Implications

This study has implications for policy, practice, and research. Since the implementation of mandatory arrest and charging policies, there has been an increase in the number of women arrested for abusing their partners (Poon et al., 2014). However, as elucidated through the narratives of the Black women in this study, these policies are flawed because they penalize women who are victims of IPV, further disempowering them. In addition, they fail to consider the complexities of violence in their lives. These criminalization strategies “deny women choice and fail to acknowledge [Black women's] multiple allegiances to themselves, to their partners, and to their communities” (Hampton et al., 2008, p. 343). Given that these policies have resulted in unintended consequences for Black women (Chesney-Lind, 2002; Pollack et al., 2005), criminal justice responses must move beyond criminalizing Black women who have been victimized. In fact, these approaches must not be the only option for women who use force in their relationships. Indeed, coordinated efforts between policy makers, community partners, and the criminal justice system are necessary to develop and implement appropriate interventions that meet Black women's unique needs.

Findings from the study also have implications for practice. Further complicating Black women's experiences of IPV is their reluctance to seek formal support (Postmus, 2015), especially those who fear they may be criminalized for their actions. Women's experiences of racism, structural inequalities, social marginalization (Block et al., 2019; Maynard, 2017), unrealistic cultural expectations, and pejorative stereotypes limit women's options to sever ties with their abusive partners. Given these findings, social service providers must remove barriers and improve the delivery of appropriate services to help break Black women's silence and increase their capacity to leave their abusive relationships. Programs and services must be embedded within Black communities and focus on both prevention and intervention. Accordingly, “such integrated interventions call for collaboration and coordination of services among professionals from multiple disciplines and agencies [and] include mental health agencies, substance abuse programs … domestic violence programs … [and] clinics providing reproductive health-care services to women” (Sabri & Gielen, 2019, p. 729). It is also necessary that social service providers receive appropriate training, so they are well equipped to support Black women. Training should inform practitioners of the various systemic barriers that amplify experiences of IPV and identify the ways in which they can support Black women to overcome these barriers.

Despite an increase in the number of women arrested for abusing their partners, there is a dearth of Canadian research that examines Black women's experiences of IPV. Their experiences are often homogenized with that of other women, with little to no discussion of the differential impact of IPV in their lives. Thus, further exploration is required that examines the various factors that complicate violence in Black women's lives and the ways in which these influence their future involvement with the law.

In the current study, women born in the Caribbean were more likely than other women to be involved in an altercation that resulted in their arrest. It is possible that the higher arrest rates among these women were due to the police's negative perception of women born in the Caribbean. Caribbean women remain an understudied group (Lacey et al., 2015); therefore, examining how police perceptions of women born in the Caribbean influence arrest decisions could better inform this area of research. While findings from this study were based on women's self-reports of their experiences of IPV, further research is required that considers the perspectives of both partners. According to Potter (2007b), including “batterers and the social, cultural, and individual sources of their behaviors will lead to a more holistic understanding of this phenomenon that continues to be shrouded in the belief that partner abuse is a private or personal matter” (p. 368). Thus, efforts must also engage men to critically reflect on their role in helping to eradicate male violence. Finally, findings from the research suggest that economic marginality exacerbated Black women's experiences of IPV; therefore, subsequent research must examine the interconnections between economic marginality and IPV for Black women.

## References

[bibr1-10778012211035791] Abraham M. Tastsoglou E. (2016). Addressing domestic violence in Canada and the United States: The uneasy co-habitation of women and the state. Current Sociology Monograph, 64(4), 568–585. 10.1177/0011392116639221

[bibr2-10778012211035791] Alliance for Heathier Communities. (2020). Letter to Premier Ford, Deputy Premier Elliott and Dr. Williams regarding the need to collect and use socio-demographic and race-based data. https://www.allianceon.org/news/Letter-Premier-Ford-Deputy-Premier-Elliott-and-Dr-Williams-regarding-need-collect-and-use-socio

[bibr3-10778012211035791] Ansara D. L. Hindin M. J. (2011). Psychosocial consequences of intimate partner violence for women and men in Canada. Journal of Interpersonal Violence, 26(8), 1628–1645. 10.1177/0886260510370600 20501897

[bibr4-10778012211035791] Bensadoun E. (2021, February 18). Over 50% of Canadians think systemic racism built into country‘s institutions, polls says. *Global News* . https://globalnews.ca/news/7632579/canada-systemic-racism-foundation/

[bibr5-10778012211035791] Bent-Goodley T. B. (2001). Eradicating domestic violence in the African American community: A literature review and action agenda. Trauma, Violence & Abuse, 2(4), 316–330. 10.1177/1524838001002004003

[bibr6-10778012211035791] Bent-Goodley T. B. (2004). Perceptions of domestic violence: A dialogue with African American women. Health and Social Work, 29(4), 307–316. 10.1093/hsw/29.4.307 15575458

[bibr7-10778012211035791] Bent-Goodley T. B. (2011). The ultimate betrayal: A renewed look at intimate partner violence. NASW Press.

[bibr8-10778012211035791] Bent-Goodley T. B. Fowler D. N. (2006). Spiritual and religious abuse: Expanding what is known about domestic violence. Affilia, 21(3), 282–295. 10.1177/0886109906288901

[bibr9-10778012211035791] Black M. C. Basile K. C. Breiding M. J. Smith S. G. Walters M. L. Merrick M. T. Chen J. Stevens M. R. (2011). The National Intimate Partner and Sexual Violence Survey (NISVS) (2010 Summary Report). National Center for Injury Prevention and Control, Centers for Disease Control and Prevention. http://www.cdc.gov/violenceprevention/pdf/nisvs_executive_summary-a.pdf

[bibr10-10778012211035791] Block S. Galabuzi G. E. Tranjan R. (2019). Canada’s colour coded income inequality. Canadian Centre for Policy Alternatives. https://www.policyalternatives.ca/publications/reports/canadas-colour-coded-income-inequality

[bibr11-10778012211035791] Brown D. (2007). Critical race theory: Case materials and problems (2nd ed.). Thomson/West.

[bibr12-10778012211035791] Brown G. (2012). Ain’t I a victim? The intersectionality of race, class, and gender in domestic violence and the courtroom. (Legal Studies Research Paper No. 12-06). Valparaiso University. 10.2139/ssrn.2050083

[bibr13-10778012211035791] Burczycka M. (2018). Section 2: Police-reported intimate partner violence in Canada, 2018. (Statistics Canada. Juristat, Catalogue no. 85-002-X). The Canadian Centre for Justice Statistics. Statistics Canada. https://www150.statcan.gc.ca/n1/pub/85-002-x/2019001/article/00018/tbl/tbl02-3-eng.htm

[bibr14-10778012211035791] Burczycka M. Conroy S. Savage L. (2018). Family violence in Canada: A statistical profile, 2017. (Statistics Canada. Juristat, Catalogue no. 85-002-X). The Canadian Centre for Justice Statistics. https://www150.statcan.gc.ca/n1/en/pub/85-002-x/2018001/article/54978-eng.pdf?st=iiI5r1cv

[bibr15-10778012211035791] Cafferky B. M. Mendez M. Anderson J. R. Stith S. M. (2016). Substance use and intimate partner violence: A meta-analytic review. Psychology of Violence, 8(1), 110–131. 10.1037/vio0000074

[bibr16-10778012211035791] Carbone-Lopez K. Kruttschnitt C. (2010). Risky relationships? Assortative mating and women’s experiences of intimate partner violence. Crime & Delinquency, 56(3), 358–384. 10.1177/0011128709333727

[bibr17-10778012211035791] Carney M. M. Buttell F. P. (2006). An evaluation of a court-mandated batterer intervention program: Investigating differential program effect for African American and white women. Research on Social Work Practice, 16(6), 571–581. 10.1177/1049731506289115

[bibr18-10778012211035791] Chan W. Chunn D. (2014). Racialization, crime, and criminal justice in Canada. University of Toronto Press.

[bibr19-10778012211035791] Chaney C. (2009). Boys to men: How perceptions of manhood influence the romantic partnerships of Black men. Western Journal of Black Studies, 33(2), 110–122.

[bibr20-10778012211035791] Chesney-Lind M. (2002). Criminalizing victimization: The unintended consequences of pro-arrest policies for girls and women. Criminology & Public Policy, 2(1), 81–90. 10.1111/j.1745-9133.2002.tb00108.x

[bibr21-10778012211035791] Collins P. H. (2000). Black feminist thought. Knowledge, consciousness, and the politics of empowerment (2nd ed.). Routledge.

[bibr22-10778012211035791] Comack E. Brickey S. (2007). Constituting the violence of criminalized women. Canadian Journal of Criminology and Criminal Justice, 49(1), 1–36. 10.3138/5523-4873-1386-5453

[bibr23-10778012211035791] Conroy S. Burczycka M. Savage L. (2019). Family violence in Canada: A statistical profile, 2018. (Juristat. Statistics Canada, Catalogue no. 85-002-X). Statistics Canada.

[bibr24-10778012211035791] Cotter A. Savage L. (2019). Gender-based violence and unwanted sexual behaviour in Canada, 2018: Initial findings from the Survey of Safety in Public and Private spaces. Statistics Canada. https://www150.statcan.gc.ca/n1/pub/85-002-x/2019001/article/00017-eng.htm

[bibr25-10778012211035791] Cranford C. J. Vosko L. F. Zukewich N. (2003). Precarious employment in the Canadian labour market: A statistical portrait. Just Labour, 3(Fall 2003), 6–22. 10.25071/1705-1436.164

[bibr26-10778012211035791] Crann S. E. Barata P. C. (2016). The experience of resilience for adult female survivors of intimate partner violence: A phenomenological inquiry. Violence Against Women, 22(7), 853–875. 10.1177/1077801215612598 26567293

[bibr27-10778012211035791] Creamer J. (2020). Inequalities persist despite decline in poverty for all major race and Hispanic origin group. United Census Bureau. https://www.census.gov/library/stories/2020/09/poverty-rates-for-blacks-and-hispanics-reached-historic-lows-in-2019.html

[bibr28-10778012211035791] Crenshaw K. (1991). Mapping the margins: Intersectionality, identity politics, and violence against women of colour. Stanford Law Review, 43(6), 1241–1299. 10.2307/1229039

[bibr29-10778012211035791] Creswell J. W. Creswell J. D. (2018). Research design. Qualitative, quantitative and mixed methods approaches (5th ed). SAGE Publishing.

[bibr30-10778012211035791] Department of Justice. (2017). Final report of the ad hoc federal-provincial-territorial working group reviewing spousal abuse policies and legislation. https://www.justice.gc.ca/eng/rp-pr/cj-jp/fv-vf/pol/p2.html

[bibr31-10778012211035791] Dobash R. P. Dobash R. E. (2004). Women’s violence to men in intimate relationships: Working on a puzzle. British Journal of Criminology, 44(3) 324–349. 10.1093/bjc/azh026

[bibr32-10778012211035791] Donovan R. Williams M. (2013). Living at the intersection: The effects of racism and sexism on Black rape survivors. In C. M. West (Ed.), Violence in the lives of Black women: Battered black and blue (pp. 169–185). Jessica Kingsley Publishers. 10.1300/J015v25n03_07

[bibr33-10778012211035791] Esqueda C. W. Harrison L. A. (2005). The influence of gender role stereotypes, the woman’s race, and level of provocation and resistance on domestic culpability attributions. Sex Roles, 53(11/12), 821–834. 10.1007/11199s-005-8295-1

[bibr34-10778012211035791] Etowa J. B. Beagan B. L. Eghan F. Thomas Bernard W. (2017). “You feel you have to be made of steel”: The strong Black woman, health, and well-being in Nova Scotia. Health Care for Women International, 38(4), 379–393. 10.1080/07399332.2017.1290099 28151098

[bibr35-10778012211035791] Few A. L. (2005). The voices of Black and white rural battered women in domestic violence shelters. Family Relations, 54(4), 488–500. 10.1111/j.1741-3729.2005.00335.x

[bibr36-10778012211035791] Fiebert M. S. (2014). References examining assaults by women on their spouses or male partners: An updated annotated bibliography. Sexuality & Culture, 18(2), 405–467. 10.1007/s12119-013-9194-1

[bibr37-10778012211035791] Gillum T. L. (2002). Exploring the link between stereotypic images and intimate partner violence in the African American community. Violence Against Women, 8(1), 64–86. 10.1177/10778010222182946

[bibr38-10778012211035791] Gillum T. L. (2008). Community response and needs of African American female survivors of domestic violence. Journal of Interpersonal Violence, 23(1), 39–57. 10.1177/0886260507307650 18087031

[bibr39-10778012211035791] Gillum T. L. (2019). African American survivors of intimate partner violence: Lived experience and future directions for research. Journal of Aggression, Maltreatment & Trauma, 1–18. 10.1080/10926771.2019.1607962

[bibr40-10778012211035791] Goodmark L. (2008). When is a battered woman not a battered woman? When she fights back. Yale Journal of Law and Feminism, 20(1), 75–129.

[bibr41-10778012211035791] Hamberger L. Larsen S. (2015). Men’s and women’s experience of intimate partner violence: A review of ten years of comparative studies in clinical samples. Part I. Journal of Family Violence, 30(6), 699–717. 10.1007/s10896-015-9732-8

[bibr42-10778012211035791] Hampton R. Oliver W. Magarian L. (2003). Domestic violence in the African American community. An analysis of social and structural factors. Violence Against Women, 9(5), 533–557. 10.1177/1077801202250450

[bibr43-10778012211035791] Hampton R. L. LaTaillade J. J. Dacey A. Marghi J. R. (2008). Evaluating domestic violence interventions for Black women. Journal of Aggression, Maltreatment & Trauma, 16(3), 330–353. 10.1080/10926770801925759

[bibr44-10778012211035791] Harrison L. A. Esqueda C. W. (1999). Myths and stereotypes of actors involved in domestic violence: Implications for domestic violence culpability attributions. Aggression and Violent Behaviours, 4(2), 129–138. 10.1016/S1359-1789(97)00026-8

[bibr45-10778012211035791] Hutchins H. Sinha M. (2013). Section 3: Impact of violence against women. Statistics Canada. https://www150.statcan.gc.ca/n1/pub/85-002-x/2013001/article/11766/11766-3-eng.htm

[bibr46-10778012211035791] Kernsmith P. (2005). Exerting power or striking back: A gendered comparison of motivations for domestic violence perpetration. Violence and Victims, 20(2), 173–185. 10.1891/vivi.2005.20.2.173 16075665

[bibr47-10778012211035791] Lacey K. K. Jiwatram-Negron T. Powell Sears K. (2021). Help seeking behaviours and barriers among Black women exposed to severe intimate partner violence: Findings from a nationally representative sample. Violence Against Women, 27(6–7), 952–972. 10.1177/1077801220917464 32498628

[bibr48-10778012211035791] Lacey K. K. Sears K. P. Matusko N. Jackson J. S. (2015). Severe physical violence and Black women’s health and well-being. American Journal of Public Health, 105(4), 719–724. 10.2105/AJPH.2014.301886 24922123 PMC4358204

[bibr49-10778012211035791] Matsuda M. J. (1989). When the first quail calls: Multiple consciousness as jurisprudential method. Women’s Rights Law Reporter, 11(1), 7–10.

[bibr50-10778012211035791] Maynard R. (2017). Policing Black lives: State violence in Canada from slavery to present. Fernwood Publishing.

[bibr51-10778012211035791] McKinney C. M. Caetano R. Rodriguez L. A. Okoro N. (2010). Does alcohol involvement increase the severity of intimate partner violence? Alcoholism: Clinical and Experiential Research, 34(4), 655–658. 10.1111/j.1530-0277.2009.01134.x PMC3280687 20102574

[bibr52-10778012211035791] Moe A. M. (2004). Blurring the boundaries: Women’s criminality in the context of abuse. Women’s Studies Quarterly, 32(3/4), 116–138.

[bibr53-10778012211035791] Moreau G. (2019). Canadian residential facilities for victims of abuse, 2017/2018. Statistics Canada. https://www150.statcan.gc.ca/n1/pub/85-002-x/2019001/article/00007-eng.htm#n26-refa

[bibr54-10778012211035791] Moss V. A. Pitula C. R. Campbell J. C. Halstead L. (1997). The experience of terminating an abusive relationship from an Anglo and African American perspective: A qualitative descriptive study. Issues in Mental Health Nursing, 18(5), 433–454. 10.3109/01612849709009423 9362722

[bibr55-10778012211035791] Nash S. T. (2005). Through black eyes: African American women’s constructions of their experiences with intimate male partner violence. Violence Against Women, 11(11), 1420–1440. 10.1177/1077801205280272 16204732

[bibr56-10778012211035791] Nowotny K. M. Graves J. L. (2013). Substance use and intimate partner violence victimization among white, African American, and Latina women. Journal of Interpersonal Violence, 28(17), 3301–3318. 10.1177/0886260513496903 23946141 PMC6252247

[bibr57-10778012211035791] O’Leary S. G. Slep A. M. S. (2006). Precipitants of partner aggression. Journal of Family Psychology, 20(2), 344–347. 10.1037/0893-3200.20.2.344 16756412

[bibr58-10778012211035791] Pollack S. (2007). I’m just not good in relationships. Victimization discourses and the gendered regulation of criminalized women. Feminist Criminology, 2(2), 158–174. 10.1177/1557085106297521

[bibr59-10778012211035791] Pollack S. Green V. Allspach A. (2005). Women charged with domestic violence in Toronto: The unintended consequences of mandatory charge policies. http://www.oaith.ca/assets/files/Publications/womenchargedfinal.pdf

[bibr60-10778012211035791] Poon J. Dawson M. Morton M. (2014). Factors increasing the likelihood of sole and dual charging of women for intimate partner violence. Violence Against Women, 20(12), 1447–1472. 10.1177/1077801214557954 25398371

[bibr61-10778012211035791] Postmus J. L. (2015). Women from different ethnic groups and their experiences with victimization and seeking help. Violence Against Women, 21(3), 376–393. 10.1177/1077801214568254 25680802

[bibr62-10778012211035791] Potter H. (2008). Battle cries. Black women and intimate partner abuse. NYU Press.

[bibr63-10778012211035791] Potter H. (2007a). Battered Black women’s use of religious services and spirituality for assistance in leaving abusive relationships. Violence Against Women, 13(3), 262–284. 10.1177/1077801206297438 17322271

[bibr64-10778012211035791] Potter H. (2007b). The need for a multi-faceted response to intimate partner abuse perpetrated by African Americans. Criminology & Public Policy, 6(2), 367–375. 10.1111/j.1745-9133.2007.00442.x

[bibr65-10778012211035791] QSR International. (n.d.). https://www.qsrinternational.com/

[bibr66-10778012211035791] Randall M. Venkatesh V. (2015). The right to no: The crime of marital rape, women’s human rights, and international law. Brooklyn Journal of International Law, 4(1), 152–202. 10.2139/ssrn.2704099

[bibr67-10778012211035791] Razack S. Smith M. Thobani S. (2010). Introduction. States of race: Critical race feminism for the 21st century. In S. Razack M. Smith& S. Thobani (Eds.), States of race: Critical race feminism for the twenty-first century (pp. 1–22). Between the Lines.

[bibr68-10778012211035791] Rennison C. Planty M. (2003). Nonlethal intimate partner violence: Examining race, gender, and income patterns. Violence and Victims, 18(4), 433–443. 10.1891/vivi.2003.18.4.433 14582864

[bibr69-10778012211035791] Richie B. E. (1996). Compelled to crime: The gender entrapment of black battered women. Routledge.

[bibr70-10778012211035791] Richie B. E. (2012). Arrested justice: Black women, violence, and American's prison nation. New York University Press.

[bibr71-10778012211035791] Ristock J. L. (2011). Introduction. Intimate partner violence in LGBTQ lives. In J. L. Ristock (Ed.), Intimate partner violence in LGBTQ lives (pp. 1–9). Routledge.

[bibr72-10778012211035791] Sabri B. Gielen A. (2019). Integrated multicomponent interventions for safety and health risks among Black female survivors of violence: A systematic review. Trauma, Violence & Abuse, 20(5), 720–731. 10.1177/1524838017730647 PMC5771976 29334001

[bibr73-10778012211035791] Sinha M. (2013). Family violence in Canada: A statistical profile, 2011. (Catalogue no. 85-002-X). Statistics Canada. https://www150.statcan.gc.ca/n1/en/pub/85-002-x/2013001/article/11805-eng.pdf?st=q2tN8dLU

[bibr74-10778012211035791] Solorzano D. G. Yosso T. J. (2002). Critical race methodology: Counter-storytelling as an analytical framework for education research. Qualitative Inquiry, 8(1), 23–44. 10.1177/107780040200800103

[bibr75-10778012211035791] Statistics Canada. (2020a, February 25). A socioeconomic portrait of Canada’s Black population. https://www150.statcan.gc.ca/n1/en/daily-quotidien/200225/dq200225b-eng.pdf?st=dnsqBCcu

[bibr76-10778012211035791] Statistics Canada. (2020b, February 25). Canada’s Black population: Education, labour and resilience. https://www150.statcan.gc.ca/n1/pub/89-657-x/89-657-x2020002-eng.pdf

[bibr77-10778012211035791] Stevens-Watkins D. Perry B. Harp K. L. Oser C. B. (2012). Racism and illicit drug use among African American women: The protective effects of ethnic identity, affirmation, and behavior. Journal of Black Psychology, 38(4), 471–496. 10.1177/0095798412438395 24482547 PMC3904443

[bibr78-10778012211035791] Stockman J. K. Lucea M. B. Bolyard R. Bertand D. Callwood G. B. Sharps P. W. Campbell D. W. Campbell J. C. (2014). Intimate partner violence among African American and African Caribbean women: Prevalence, risk factors, and the influence of cultural attitudes, Global Health Action, 7(1), 24772. 10.3402/gha.v7.24772 25226418 PMC4165044

[bibr79-10778012211035791] Straus M. A. (2010). Thirty years of denying the evidence on gender symmetry in partner violence: Implications for prevention and treatment. Partner Abuse, 1(3), 332–362. 10.1891/1946-6560.1.3.332

[bibr80-10778012211035791] Stuart G. L. Temple J. R. Follansbee K. W. Bucossi M. M. Hellmuth J. C. Moore T. M. (2008). The role of drug use in a conceptual model of intimate partner violence in men and women arrested for domestic violence. Psychology of Addictive Behaviors, 22(1), 12–24. 10.1037/0893-164X.22.1.12 18298227

[bibr81-10778012211035791] St. Vil V. M. Sabri B. Nwokolo V. Alexander K. A. Campbell J. C. (2017). A qualitative study of survival strategies used by low-income Black women who experience intimate partner violence, Social Work, 62(1), 63–71, 10.1093/sw/sww080 28395046 PMC5391770

[bibr82-10778012211035791] Taft C. T. Bryant-Davis T. Woodward H. E. Tillman S. Torres S. E. (2009). Intimate partner violence against African American women: An examination of the socio-cultural context. Aggression and Violent Behavior, 14(1), 50–58. 10.1016/j.avb.2008.10.001

[bibr83-10778012211035791] Taylor J. Y. (2005). No resting place: African American women at the crossroads of violence. Violence Against Women, 11(12), 1473–1489. 10.1177/1077801205280275 16247112

[bibr84-10778012211035791] Taylor J. Y. (2008). The straw that broke the camel’s back. Women & Therapy, 25(3–4), 79–94. 10.1300/J015v25n03_06

[bibr85-10778012211035791] Truman J. L. Morgan R. E. (2014). Nonfatal domestic violence, 2003–2012. Bureau of Justice Statistics, US Department of Justice, Office of Justice Programs. https://www.ncjrs.gov/App/Publications/abstract.aspx?ID=266778

[bibr86-10778012211035791] Ullman S. E. Lorenz K. (2020). African American sexual assault survivors and mental health help-seeking: A mixed methods study. Violence Against Women, 26(15–16), 1941–1965. 10.1177/10778012198926501077801219892650 31896312 PMC7332382

[bibr87-10778012211035791] Ward R. E. Jr. Muldoon J. P. (2007). Female tactics and strategies of intimate partner violence: A study of incident reports. Sociological Spectrum, 27(4), 337–364. 10.1080/02732170701290985

[bibr88-10778012211035791] Watson N. N. Hunter C. D. (2016). “I had to be strong”: Tensions in the strong Black woman schema. Journal of Black Psychology, 42(5), 424–452. 10.1177/0095798415597093

[bibr89-10778012211035791] West C. M. (2004). Black women and intimate partner violence: New directions for research. Journal of Interpersonal Violence, 19(12), 1487–1493. 10.1177/0886260504269700 15492062

[bibr90-10778012211035791] West C. M. (2005). Domestic violence in ethnically and racially diverse families: The “political gag order” has been lifted. In N. J. Sokoloff & C. Pratt (Eds.), Domestic violence at the margins: A reader at the intersection of race, class, and gender (pp. 157–173). Rutgers University Press.

[bibr91-10778012211035791] West C. M. (2007). “Sorry, we have to take you in:” Black battered women arrested for intimate partner violence. Journal of Aggression, Maltreatment & Trauma, 15(3–4), 95–121. 10.1080/10926770802097277

[bibr92-10778012211035791] West C. M. (2012). Mammy, jezebel, sapphire, and their homegirls: Developing an “oppositional gaze” toward the images of black women. In J. C. Chrisler C. Golden & P. D. Rozee (Eds.), Lectures on the psychology of women (4th ed., pp. 286–299). Waveland Press, Inc.

[bibr93-10778012211035791] Wing A. K. (Ed.). (2003). Critical race feminism: A reader (2nd ed.). New York University Press.

[bibr94-10778012211035791] World Health Organization. (2021). Violence against women. https://www.who.int/news-room/fact-sheets/detail/violence-against-women

